# Evolution of anti-malaria policies and measures in P.R. China for achieving and sustaining malaria-free

**DOI:** 10.3389/fpubh.2023.1094859

**Published:** 2023-03-03

**Authors:** Jian-Hai Yin, Li Zhang, Xin-Yu Feng, Zhi-Gui Xia

**Affiliations:** ^1^National Institute of Parasitic Diseases, Chinese Center for Disease Control and Prevention (Chinese Center for Tropical Diseases Research), Shanghai, China; ^2^NHC Key Laboratory of Parasite and Vector Biology, Shanghai, China; ^3^WHO Collaborating Center for Tropical Diseases, Shanghai, China; ^4^National Center for International Research on Tropical Diseases, Shanghai, China

**Keywords:** malaria, control and elimination, policy, strategy and measure, China

## Abstract

Malaria is a major public health threat worldwide, and it was also widely prevalent in the history in China, seriously endangering people's health and affecting socioeconomic development. China was certified malaria elimination in 2021 with unremitting efforts since the founding of the People's Republic of China in 1949. This great achievement has been another milestone in the fight against major infectious diseases following the elimination of smallpox, poliomyelitis, leprosy, filariasis, neonatal tetanus and blinding trachoma in China. This paper briefly introduces the malaria burden dynamics and the corresponding malaria transmission risk stratificantions, as well as systematically reviews the evolution of anti-malaria policies and measures from severe epidemic to elimination in China. Meanwhile, five key lessons in malaria control and elimination in China are also briefly summarized. All of the above provide evidences for promoting global malaria eradication and preventing reestablishment of malaria transmission, finally benefit all individuals still suffering from the scourge of malaria.

## Introduction

Malaria is a serious and even fatal disease caused by *Plasmodium* parasites that are transmitted to people through the bites of infected female *Anopheles* mosquitoes. *Plasmodium* parasites that infect humans include *Plasmodium falciparum, P. vivax, P. malariae, P. ovale* (including *P. ovale curtisi* and *P. ovale wallikeri*) and *P. knowlesi*, of which *P. falciparum* is the most lethal, and *P. vivax* and *P. ovale* can relapse due to the presence of latent liver stages known as hypnozoites, which can activate weeks, months, or even years after the primary clinical infection (1, 2).

Although more and more countries were certified as malaria-free or approaching to malaria elimination (3), malaria is still one of the major public health threats worldwide, nearly half of the world's population was at risk of malaria with around 247 million cases of malaria and 619,000 malaria deaths in 2021, and infants, children under 5 years of age, pregnant women and those with low immunity are the most vulnerable populations (4).

In China, malaria can be dated back to about 3,000 years ago, and spread widely in the history, seriously endangering people's health and affecting socioeconomic development. Before 1949, it was estimated that more than 350 million of the total population of about 450 million were threatened by malaria, and there were at least 30 million malaria patients every year, with a fatality of about 1% (5). After the founding of the People's Republic of China (P.R. China), the national malaria burden has been greatly reduced with several generations of continuous efforts in the fight against malaria (6, 7), and the last indigenous case was reported in April 2016 (8, 9). China officially submitted an application for malaria elimination certification to the WHO in November 2020, and was certified as malaria-free on June 30, 2021 (10, 11), which has been a great milestone in the history of China's fight against major infectious diseases, and is bound to have an important impact on the development of China's public health. However, China still faces many challenges in the maintenance of malaria-free status (12). Therefore, we systematically review the anti-malaria history in the past decades from the perspective of evolution of policies and measures in China, in order to provide experiences of malaria control and elimination for promoting global malaria eradication and preventing reestablishment of malaria transmission in China.

## Brief of malaria transmission in P.R. China

### Dynamics of malaria burden

**Figure 1:**
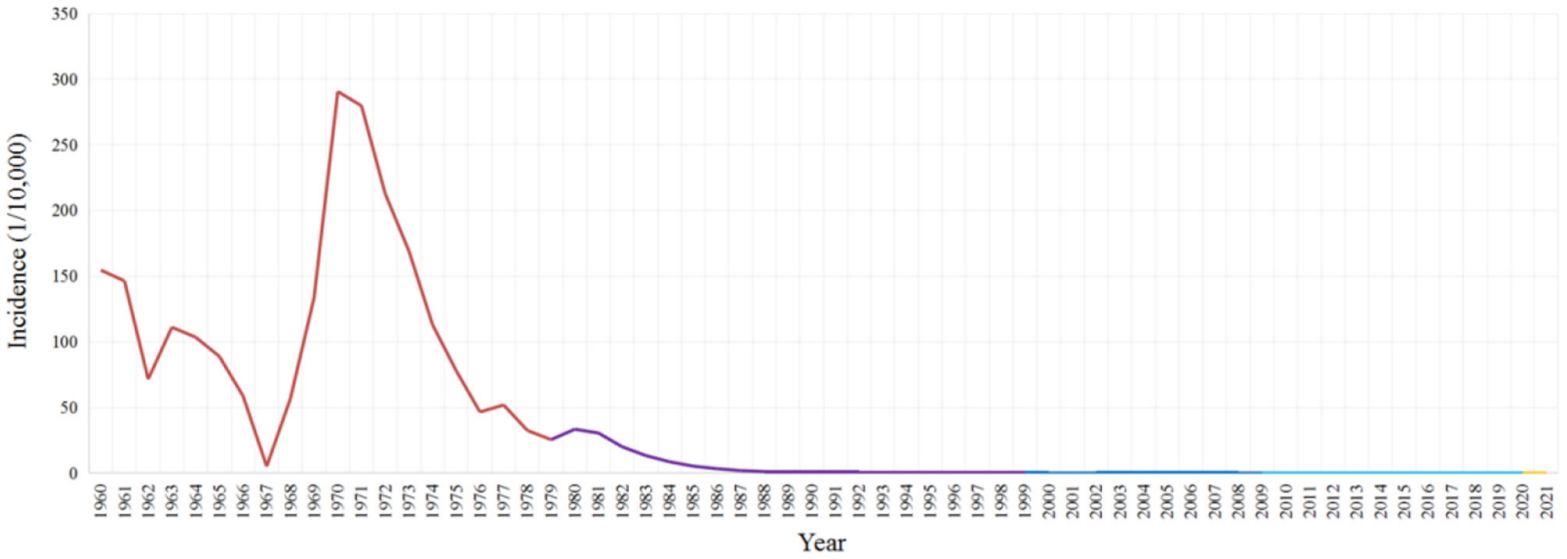
Incidence of indigenous malaria reported in China, 1960–2021. There was a lack of systematic records for malaria cases from 1950 to 1959, and the incidence of indigenous malaria reported has remained zero since 2017.

According to epidemic statistics, about 228 million malaria cases and about 36,000 malaria deaths were reported in the Chinese mainland (excluding Taiwan, Hong Kong and Macao) from 1950 to 2021 (Figure 1). Furthermore, malaria transmission was serious from the 1950s to 1980s. About 32 million cases and 26,000 deaths from 1950 to 1959, and about 69 million cases and 6,000 deaths from 1960 to 1969, and about 114 million cases and 2,400 deaths from 1970 to 1979, and about 12 million cases and 500 deaths from 1980 to 1989, were reported respectively. And it reached to the peak in 1970 with more than 24 million cases nationwide, and 91.2% of which were reported in five provinces (Jiangsu, Shandong, Henan, Anhui, and Hubei), and two large-scale outbreaks were occurred around 1960 and 1970 separately (7, 13). In addition, about 600,000 cases and 400 deaths were reported from 1990 to 1999, then the malaria transmission rebounded and local outbreaks were occurred in Anhui and some other provinces from 2001 to 2006 (14), and the number of malaria cases nationwide reached 64,178 cases in 2006 (15), and about 350,000 cases and about 300 deaths were totally reported from 2000 to 2009 (6, 15–23).

In order to actively response to the health-related Millennium Development Goals, and the ultimate global goal of malaria eradication, the Chinese government launched the national malaria elimination program (NMEP) in 2010, with the corresponding National Action Plan for Malaria Elimination in China (2010–2020) (24). As a result, no indigenous cases have been reported since 2017 (25), although there were about 38,000 cases and <200 deaths reported from 2010 to 2021, as well as thousands of imported cases reported each year (8, 25–35).

**Figure 2:**
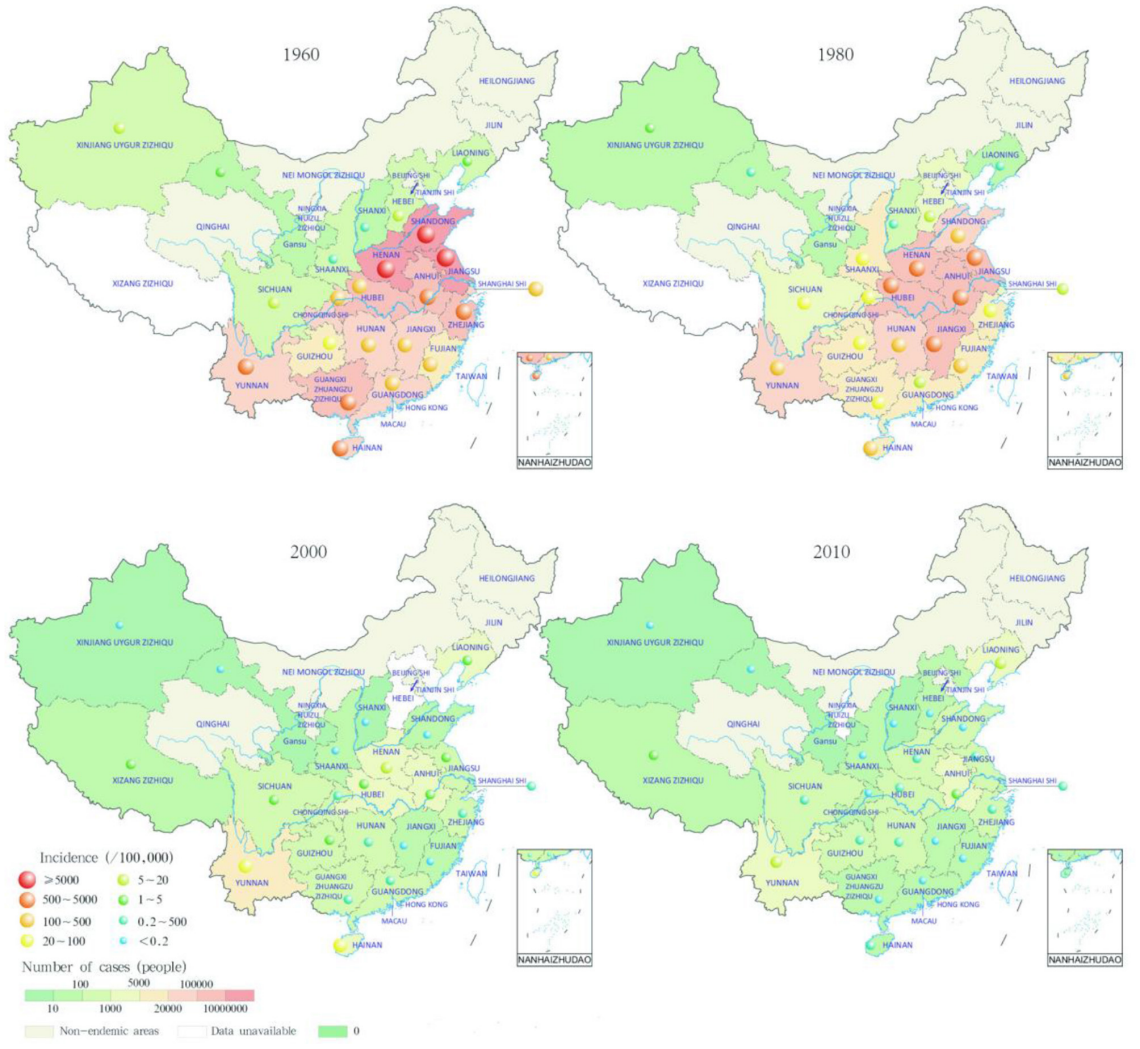
Trends of malaria cases reported in China. Malaria cases reported in the first year of the control of severe epidemics stage (1960–1979), the incidence decline stage (1980–1999), the achievement consolidation/pre-elimination stage (2000–2009), and the elimination phase (2010–2020), respectively (36).

With the launch of the NMEP in 2010 and the WHO certification of malaria-free in 2021 as milestones, three phases of the malaria control phase (1949–2009), the elimination phase (2010–2020) and the post-elimination phase (2021~) can be divided from the founding of the People's Republic of China. Furthermore, the control phase could be roughly divided into four different stages according to the characteristics of malaria transmission and the anti-malaria policies and measures in different periods, namely, the focal investigation and control stage (1949–1959), the control of severe epidemics stage (1960–1979), the incidence decline stage (1980–1999), and the achievement consolidation/pre-elimination stage (2000–2009) (13) (Figure 2).

### Stratifications of malaria-endemic areas

In the early days of the founding of the People's Republic of China, it was estimated that there were 1,829 malaria-endemic counties nationwide, accounting for more than 70% of the total number of counties at that time (37). The classification of malaria-endemic areas was often associated with reported cases and local distribution of *Anopheles* mosquitoes. It was recorded that four *Plasmodium* species (*P. falciparum, P. vivax, P. malariae, P. ovale*) have been endemic in China, among which *P. vivax* and *P. falciparum* were the main species. Moreover, *P. vivax* was distributed nationwide, and *P. falciparum* was the dominant species in the hyperendemic area in southern China, which was the major cause of severe malaria and death. In addition, *P. malariae* was mainly scattered in southern China, and *P. ovale* was only found sporadically in some areas of southwest China and some counties in Hainan.

**Figure 3:**
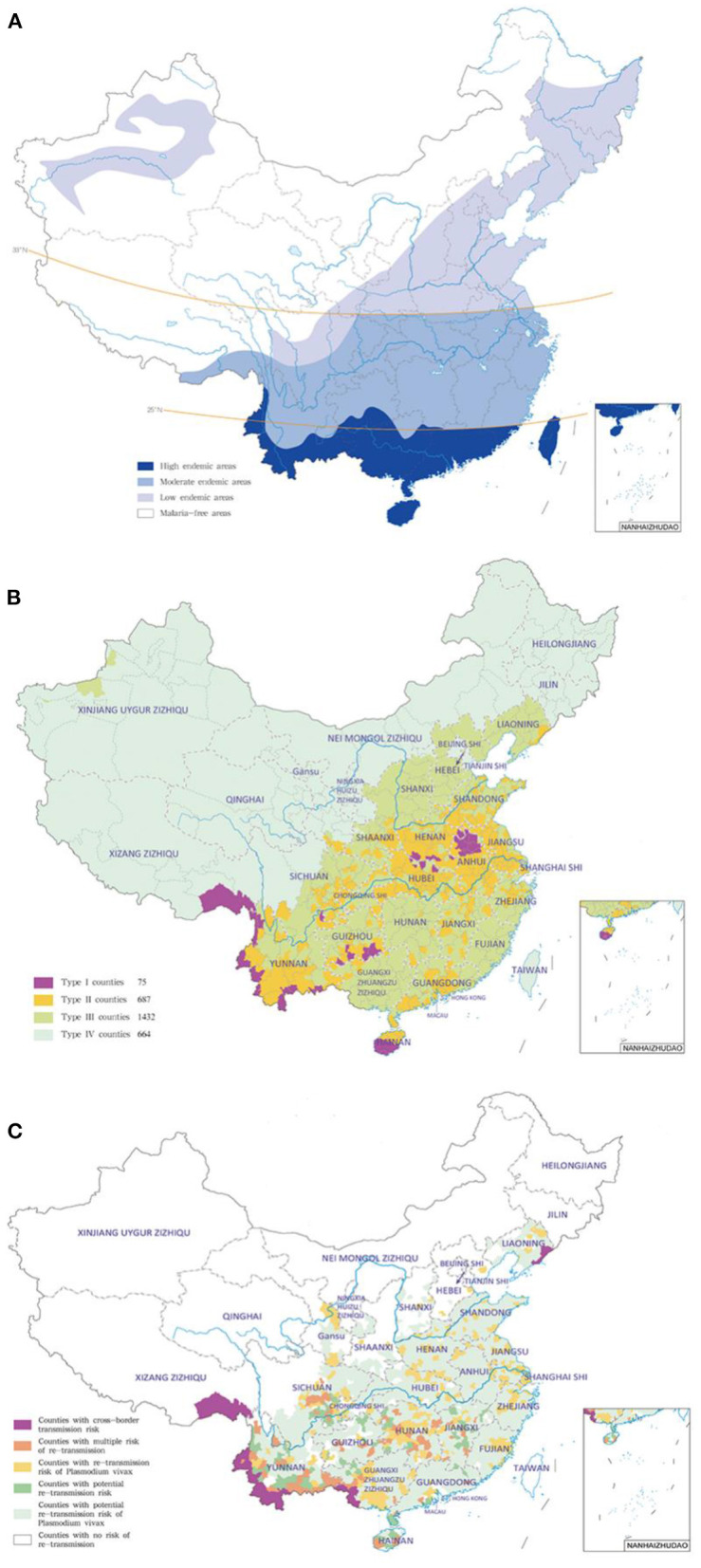
Representative stratification of malaria-endemic areas in different periods (36). **(A)** Control phase; **(B)** elimination phase; **(C)** post-elimination phase.

Overall, malaria prevalence was increasing from north to south in China. Four categories of malaria-endemic areas (north of 32°N, 25°-32°N, south of 25°N, and northwest) were first divided in 1958 according to the disease survey data combined with topography, climate and other factors, then this stratification was updated with more characteristics in 1965 (5). Furthermore, the latitude of 33°N was widely accepted as the north-south dividing line of malaria transmission in China, and malaria-endemic areas (north of 33°N, 25°-33°N, south of 25°N, and northwest) were classified again based on the northern margin of the distribution of *Anopheles anthropophagus* and *Anopheles minimus*, and the northern edge of *P. falciparum* malaria transmission (38). First, in the north of latitude 33°, malaria transmission was unstable and low, it was mainly distributed in the low-lying areas close to rivers, lakes and rice-growing areas, where only *P. vivax* malaria was transmitted in 3–6 months per year, and *An. sinensis* was the only malaria vector. However, *P. vivax* was the dominated species but with *P. falciparum* and *P. malariae* occasionally in the Yili River Valley in Xinjiang, and malaria vector was *An. messeae* in the north and *An. sacharovi* in the south. Second, in the area of 25°-33°N, malaria transmission was also unstable at medium or low levels with a period of 6–8 months, and *P. vivax, P. falciparum*, and *P. malariae* were all distributed, among which *P. vivax* was the mainstay, but the peak season was mostly caused by *P. falciparum*, and the main malaria vectors were *An. anthropophagus* and *An. sinensis*. Third, in the south of latitude 25°N, malaria transmission was high with a period up to 9–12 months, and *P. falciparum* was the most common species followed by *P. vivax*, while *P. malariae* was scattered, and *P. ovale* was distributed in Yunnan border and Hainan Island and some other places. Meanwhile, mixed infections were common in this area, and *An. minimus* was the main malaria vector in mountainous areas, *An. sinensis* in plain areas, and *An. dirus* in the forest of Hainan, respectively. Fourth, the desert arid areas of northwest and north China, as well as the alpine areas of southwest China and the mountainous areas of north China, are naturally malaria-free (Figure 3A).

To ensure the successful implementation of NMEP, four types of counties (Type I: indigenous malaria cases detected in 3 consecutive years and the annual incidences ≥1/10,000; Type II: indigenous malaria cases detected in the last 3 years and at least in 1 year the annual incidence <1/10,000 and >0; Type III: no indigenous malaria cases reported in the 3 years; Type IV: non-malaria endemic area) were stratified based on the national malaria reports from 2006 to 2008 in China (6, 24, 39) (Figure 3B).

In the post-elimination phase, risk stratification of the re-introduction was defined in the Technical Guideline for Prevention of Reestablishment of Malaria Transmission based on the border malaria status, the receptivity and vulnerability (40) (Figure 3C).

With the continuous advancement of malaria prevention and control in China, the incidence of malaria has dropped significantly, and the distribution range of malaria parasites has gradually shrunk. In 1962, the last indigenous case infected with *P. ovale* was reported in Pingba County in Guizhou Province (41). In 2015, the last indigenous case infected with *P. malariae* was reported in Sanya in Hainan Province (42), and the last indigenous case caused by *P. falciparum* was reported in Cangyuan County in Yunnan Province (43). In 2016, the last indigenous case of *P. vivax* malaria was reported in Yingjiang County in Yunnan Province (8, 9). However, there were still reports of recrudescent *P. malariae* malaria with long incubation period in Guangdong Province (30, 34), and thousands of imported cases caused by different *Plasmodium* species were reported in recent years (25, 28, 30, 32, 34).

## Evolution of China's anti-malaria policies and measures

**Figure 4:**
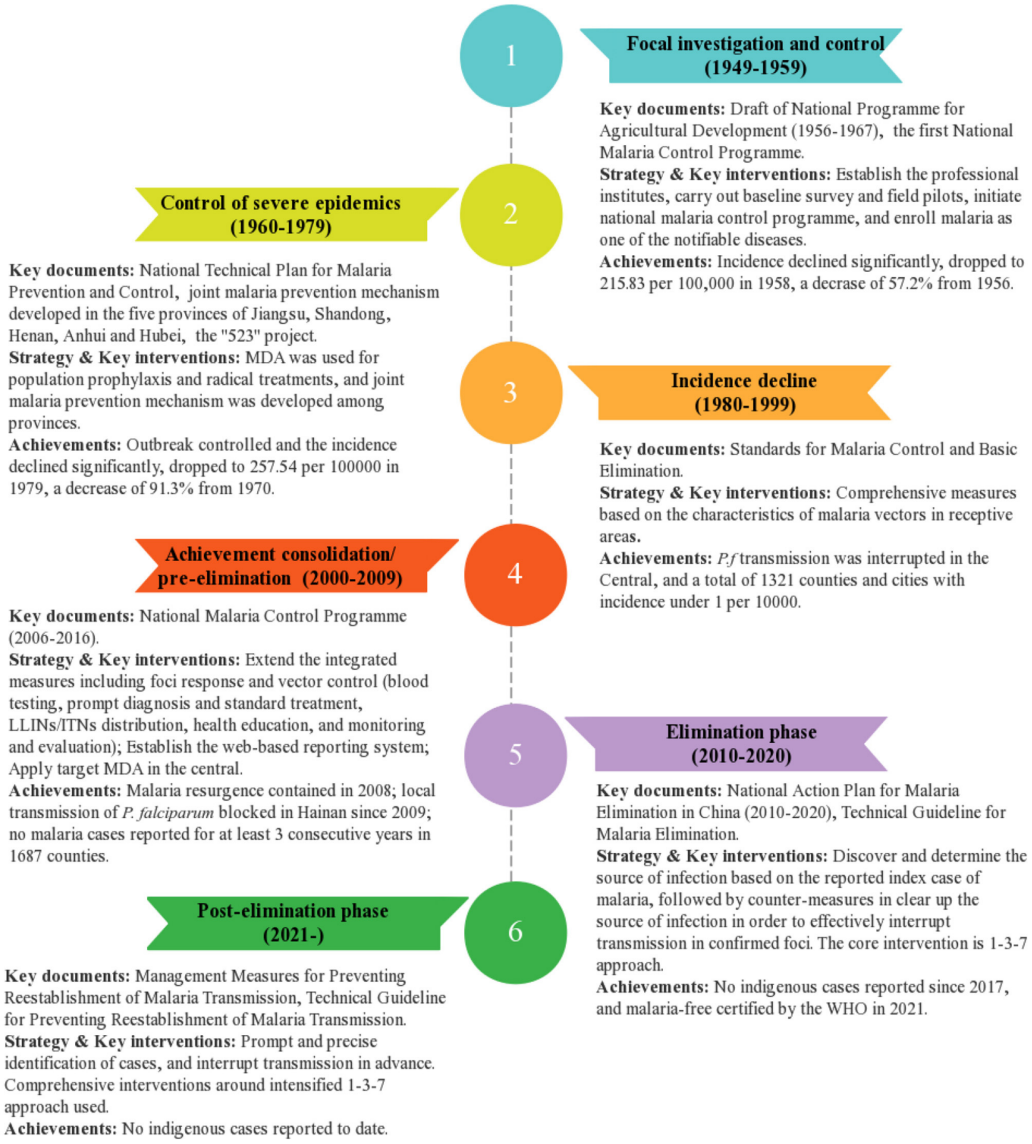
Evolution of antimalarial policies and measures from the founding of the People's Republic of China.

Different anti-malaria policies and measures were developed and implemented in the different phases (Figure 4).

### Focal investigation and control stage (1949–1959)

At this stage, several national malaria prevention and control plans and technical guidelines were launched mainly based on the focal investigation by the professional teams in the key malaria-endemic areas (5, 7, 13, 38).

In 1951, the Work Plan for the Prevention and Control of Malaria in Ethnic Minority Areas was issued. In 1952, malaria control centers (stations) responsible for malaria investigation, personnel training and pilot prevention and control were began to be established in severely endemic areas. In 1953, the report on the National Health Administrative Conference and the second National Health Conference was approved by the Government Council, and the prevention and control against the most serious endemic diseases including malaria was required to be strengthened. By 1955, malaria transmission nationwide was basically clear, and more lessons on malaria control were summarized. At the same time, a variety of antimalarial drugs such as proguanil, cycloguanil, chloroquine, primaquine and pyrimethamine could be synthesized, with a capacity to provide batch supply of some antimalarial drugs such as proguanil and cycloguanil. In 1956, the Draft of National Program for Agricultural Development (1956–1967) was proposed, in which malaria was included as one of the most serious diseases affecting the population that could be virtually eliminated in all possible areas and within a limited period of time. In the same year, the first National Malaria Control Program was developed by the Ministry of Health, and malaria was classified as a notifiable disease for the first time. In 1958, Kaili County in Guizhou Province was selected as the first pilot county for malaria eradication by the Ministry of Health.

As a result, remarkable progress such as the significant reductions of prevalence and incidence of malaria was achieved in the hyperendemic areas such as Simao County in Yunnan, Kaili County in Guizhou, and Hainan Island.

### Control of severe epidemics stage (1960–1979)

At this stage, China's malaria control unfortunately failed to achieve continuous results, and serious epidemics caused by *P. vivax* were occurred mainly in the Huanghuai Plain in the early 1960s and early 1970s.

Since 1963, chloroquine, primaquine, pyrimethamine and other key antimalarial drugs have been fully supplied, and different countermeasures in different areas according to the *Plasmoduim* species and the transmission level were specified in the National Technical Plan for Malaria Prevention and Control issued in 1964 (44), especially the radical treatment was used. In particular, malaria control had attracted great attention from the central leadership, three hyperendemic provinces of Hebei, Shandong and Henan were first jointly cooperated to fight against malaria under the direction of Premier En-lai Zhou in 1964, then Jiangsu and Anhui provinces were also included in 1965. Meanwhile, a series of measures including standardized treatment and radical cure to clear all parasites at blood stage or liver stage, prophylaxis for population in the transmitting season, and massive vector control, had been implemented, so that the incidence of malaria in the country dropped sharply year after year from 1964 to 1967. In addition, the outbreak in the 1970s had been effectively controlled with the implementation of a series of measures for prevention (radical treatment for all individuals with relapse potential, prophylaxis for the whole population in the transmitting season, prophylaxis for population in the transmitting season during the transmission peak), treatment (radical treatment, standardized treatment of patients with current symptoms, investigation and treatment of fever patients), and vector control, etc., which were adopted by the joint malaria prevention mechanism developed in the five provinces of Jiangsu, Shandong, Henan, Anhui and Hubei in 1973 (45).

During this period, in order to response to the drug resistance in malaria, more than 60 scientific research institutes and more than 500 researchers across the country were jointly organized to carry out research on malaria prevention and control, especially the “523” project (46–48), and successfully developed a batch of new antimalarial drugs such as artemisinin and its derivatives, pyronaridine phosphate, and naphthoquine phosphate.

### Incidence decline stage (1980–1999)

At this stage, comprehensive malaria control measures were applied mainly according to the characteristics of malaria transmission in different areas with different malaria vectors and their biological characteristics (13, 49–52). First, in areas with the distribution of *An. sinensis*, measures were focused on the prevention and control of infectious sources, and supplemented by vector control. Second, in areas with *An. minimus* or *An. anthropophagus*, on the contrary, measures were focused on vector control, and combined with the prevention and control of infectious sources. Third, in areas with *An. dirus*, it was also necessary to strengthen environmental transformation and reduce mosquitoes breeding sites. Moreover, in areas where the incidence has dropped below 5 per 10,000, surveillance was strengthened and residual foci needed to be removed.

In 1983, the definitions and indicators for malaria “control” and “basic elimination” were first put forward in the national malaria control program (49), followed by relevant management requirements and assessment standards (53, 54). During this period, malaria control continued to be carried out effectively, especially the malaria microscopy system for febrile patient and surveillance system were further improved, and pyrethroid insecticide-treated mosquito nets were widely used in addition to the use of insecticides such as Dichlorodiphenyltrichloroethane for indoor residual spraying to kill mosquitoes. Furthermore, the joint prevention and control mechanism for malaria control has been continuously consolidated. For example, the joint malaria management mechanism for the mobile population in the three provinces of Guangdong, Guangxi and Hainan, was formally established in 1992 (55).

As a result, the incidence of malaria continued to decline nationwide, *P. falciparum* malaria was eliminated in the central region after 1991 (56), and this species was only prevalent in Yunnan and Hainan after 1995 (57). By 1999, a total of 1,321 counties (cities) in the country had reached the standard of “basic elimination” after assessment (the annual incidence of malaria in the county was <1/10,000 for three consecutive years) (58).

### Achievement consolidation/pre-elimination stage (2000–2009)

In order to reduce the malaria transmission in Yunnan and Hainan provinces in the south, and contain the resurgence of malaria in the central, the central and local governments have increased investment in malaria control, as well as multiple rounds of programs were supported from the Global Fund since 2002 (59). In 2004, an online reporting system for infectious disease surveillance named China Information System for Disease Control and Prevention was launched nationwide (60). In 2007, the intensive measure of focal mass drug administration for populations was implemented in Anhui Province (61).

As a result, the resurgence of malaria transmission nationwide and localized outbreaks were effectively contained in 2008 (21). In 2009, Hainan Province reported its last indigenous *P. falciparum* malaria case (62), the incidence of malaria nationwide further declined, and the incidence exceeding 1/1,000 was only reported in 4 counties in the country (23). Moreover, malaria elimination pilot projects in 12 counties in 6 provinces (autonomous regions) including Hebei, Shanghai, Zhejiang, Guangdong, Fujian and Shandong were launched by the Ministry of Health at the end of the year, to explore the models and experience for malaria elimination in advance.

### Elimination phase (2010–2020)

In 2010, the National Action Plan was jointly issued by 13 ministries and commissions at the central level, which proposed to achieve the goal of malaria elimination nationwide by 2020, and the classified interventions for the stratified four types of counties (24). In 2011, the Technical Guideline for Malaria Elimination (2011 Edition) was issued by the Chinese Center for Disease Control and Prevention (CDC) (39), which further clarified the technical specifications for malaria elimination around the core of 1-3-7 approach (63): cases reported within 24 h after diagnosis, case epidemiological investigation within 3 days, and foci investigation and disposal within 1 week. In the same year, another web-based reporting system for malaria elimination program was established (64). Furthermore, an early alerting function for single case and case clusters was added for malaria automatic early warning in 2012. In order to strengthen the quality assurance of malaria testing and diagnosis nationwide, a malaria diagnostic reference laboratory network covering all 24 malaria-endemic provinces was gradually established from 2011 to 2016 (65, 66). Since 2013, the malaria elimination assessment at the county and prefectural levels has been launched, and a subnational verification of malaria elimination in all malaria-endemic provinces was carried out from 2017 to 2020 (67). In addition, a joint malaria prevention and control mechanism in the central (Shandong, Jiangsu, Anhui, Hubei, Henan), southeastern (Jiangxi, Shanghai, Zhejiang, Fujian, Hunan, Chongqing), southern (Guangxi, Guangdong, Hainan, Sichuan, Guizhou, Yunnan, Xizang) and northern (Liaoning, Hebei, Shanxi, Shaanxi, Gansu, Xinjiang) respectively was established in 2017, to consolidate the achievements of malaria elimination in each province (68).

No indigenous malaria cases were reported in the country from 2017 to 2020 (25, 28, 30, 32), and the goal of malaria elimination set by NMEP was achieved as planned. The WHO declared malaria-free in China on June 30, 2021 (10, 11).

### Post-elimination phase (2021)

Although China has achieved its malaria elimination goals, malaria vectors have not been eliminated, and there are the risk of reestablishment of transmission due to high imported malaria burden especially the border malaria in the China-Myanmar border in Yunnan Province (69–72).

In order to prevent the reestablishment of malaria transmission, the “Management Measures for Preventing Reestablishment of Malaria Transmission” was issued at the end of 2020 (73), followed by the Technical Guideline for Preventing Reestablishment of Malaria Transmission (40). Both documents aim to further strengthening surveillance and response as the core means, and remind avoiding the dilution of malaria vigilance, the reduction of financial investment, and the weakening of capacity building. It emphasizes that continued training for health personnel at all levels, multi-departmental, cross-regional and cross-border joint malaria prevention and control for risk groups in entry and exit, border and other risk areas, as well as malaria management services on education, protection, monitoring and tracking, should be carried out. Thus, it can be successful to timely detect and standardize the treatment of each imported case, and timely discover and scientifically clear-up every potential focus, thereby preventing the reestablishment of transmission, reducing severe malaria and death, and consolidating the achievements of malaria elimination.

## Lessons learned from malaria control and elimination in China

In the past more than 70 years of anti-malaria battle, China has experienced the process from 30 million to zero indigenous malaria cases with different epidemiological and socioeconomic characteristics. In general, the key lessons in malaria control and elimination in China can be briefly summarized in the following aspects.

### Strong and sustained political commitment and action

Since the founding of the People's Republic of China, governments at all levels have attached great importance to malaria control and elimination, providing sufficient organizational guarantees, and have always incorporated malaria prevention and control into the health and socioeconomic development. Large-scale and sustained malaria control and elimination programs have been organized and carried out nationwide. Moreover, sufficient technical supports from the building of professional teams and guidance of multi-level full-time personnel promoted the malaria control and elimination. In addition, these programs were supported by sufficient funding from central finance, local finance, and external funds, which laid a solid foundation for China to achieve its malaria elimination goal on schedule.

### Solid health system with a stable malaria control team

Professional personnel for epidemiological investigation and response, laboratory testing and clinical treatment are the basic force for malaria control and elimination, whether the malaria prevention and control station (institute) established in the early days of the founding of the People's Republic of China to carry out focal investigation and control, or the malaria surveillance network relying on three-level primary health network at the county, town and village levels, or the implementation of various control and elimination policies and measures, all of which are inseparable from a stable malaria control team. In addition to natural factors, the repeated recurrences of malaria transmission in China in the past were related with brain drain, team dispersion, and work stagnation. Meanwhile, the establishment of a real-time surveillance and response system with detailed case epidemiological information, the establishment of a nationwide malaria diagnostic reference laboratory network and quality management system, as well as the continuous implementation of various forms of training and assessment, have improved the level and quality from timely detection and standardized treatment of cases to rapid identification and effective response of epidemics, laying a solid foundation for malaria control and elimination.

### Scientific and reasonable strategies and targeted interventions

Development and implementation of evidence-based strategies and policies across the whole country have been the key to China's successful response to the disease. There are great differences in various topography, climate, level of socioeconomic development, people's living habits, transmission characteristics of *Plasmodium* species, distribution and ecological habits of malaria vectors, etc. in China, thus different strategies and measures have been developed, tailored and implemented in various malaria transmission phases and settings, which made control and elimination feasible in different subregions of China. For instance, the technical specifications for malaria elimination around the core of 1-3-7 approach was carried out in the elimination phase, and the strategy for prevention of malaria re-establishment was updated in a timely manner from the elimination strategy focusing on each case/focus to the prevention of reestablishment focusing on timely identification of the source of infection in the post-elimination phase.

### Active community involvement and domestic and international cooperation

Community involvement has played an important role in malaria control and elimination in China from the following three aspects at least: (1) extensive participation in health education and promotion organized by different sectors to improve public awareness and behavior of malaria prevention and control and to create a healthy and harmonious community; (2) extensive mobilization of community residents to protect environment and implement vector control in combination with new rural construction and China Patriotic Health Campaign; and (3) assistance and cooperation with local CDCs and township health centers in malaria case epidemiological investigation, foci investigation and disposal. Furthermore, inter- or multi-sectoral collaboration to develop and perform guidelines, policies, plans and measures for malaria elimination, intranational collaboration such as the joint prevention and control mechanism for malaria control in Jiangsu, Shandong, Henan, Anhui and Hubei, and the international cooperation such as the close collaboration and cooperation with the WHO, the Global Fund, and the cross-border cooperation under bilateral and multilateral mechanisms, *etc*., have not only provided advanced concepts, technologies and financial support, but also strengthened timely information sharing and experience exchange, which facilitated the malaria elimination.

### Unremitting scientific and technological innovation for malaria control and elimination

Research in basic science and field applications has made great contributions to malaria elimination in China, not only in the control and elimination strategies, but also in key technologies, covering pathogen biology, vector biology, and interventions such as diagnostics, antimalarial drugs, insecticide-treated mosquito nets and radical treatment of vivax malaria, etc. (74). Among them, the discovery of artemisinin in China which led to the Nobel Prize being awarded to Dr. Tu Youyou in 2015, reflects the contribution of Chinese traditional medicine to human health (47). Moreover, the 1-3-7 approach has been adopted by the WHO to guide elimination activities in recent years (75).

## Significance and perspectives

Malaria is another major infectious disease eliminated in China after the elimination of smallpox, poliomyelitis, leprosy, filariasis, neonatal tetanus and blinding trachoma (76), which is an important milestone in the history of the development of public health in the country, especially during the pandemic of the COVID-19. China was also the first country in the WHO Western Pacific region to be certified malaria-free since 1987 (11), marking that the country with the largest population, the longest land border and the largest number of neighboring countries has achieved the goal of eliminating malaria, which not only greatly compresses the global malaria map, but also strongly boosts the confidence of the international community to promote malaria elimination (77). We hope that the experiences from the history of malaria control and elimination in China combined with successful elimination experience in other countries will further support the global malaria elimination program, especially the E-2025 initiative (78), thus benefit all individuals still suffering from the scourge of malaria.

## Author contributions

Z-GX: conception or design of the work. J-HY and Z-GX: drafting the article. LZ and X-YF: critical revision of the manuscript. All authors final approval of the manuscript.
